# Malaria prevention practices and associated environmental risk factors in a rural community in Wakiso district, Uganda

**DOI:** 10.1371/journal.pone.0205210

**Published:** 2018-10-09

**Authors:** David Musoke, George Miiro, Rawlance Ndejjo, George Karani, Keith Morris, Simon Kasasa, Jessica Nakiyingi-Miiro, David Guwatudde, Miph Boses Musoke

**Affiliations:** 1 Department of Disease Control and Environmental Health, School of Public Health, Makerere University College of Health Sciences, Kampala, Uganda; 2 Uganda Virus Research Institute, Entebbe, Uganda; 3 Cardiff School of Health Sciences, Cardiff Metropolitan University, Cardiff, Wales, United Kingdom; 4 Department of Epidemiology and Biostatistics, School of Public Health, Makerere University College of Health Sciences, Kampala, Uganda; 5 Medical Research Council, Uganda Virus Research Institute, Entebbe, Uganda; 6 School of Sciences, Nkumba University, Entebbe, Uganda; Instituto Rene Rachou, BRAZIL

## Abstract

**Background:**

Besides use of insecticide-treated mosquito nets (ITNs) and indoor residual spraying (IRS), other complimentary measures including suitable housing structures, and environmental management that reduce breeding of malaria vectors, can be implemented at households to prevent the disease. However, most studies on malaria prevention have focused mainly on ITNs and IRS. The aim of this study was therefore to assess malaria prevention practices beyond ITNs and IRS, and associated environmental risk factors including housing structure in rural Wakiso district, Uganda.

**Methods:**

A clustered cross-sectional survey was conducted among 727 households in Wakiso district. Data were collected using an interviewer-administered questionnaire and observational checklist. The questionnaire assessed participants’ household practices on malaria prevention, whereas the checklist recorded environmental risk factors for malaria transmission, and structural condition of houses. Poisson regression modeling was used to identify factors associated with use of mosquito nets by households.

**Results:**

Of the 727 households, 471 (64.8%) owned at least one mosquito net. Use of mosquito nets by households was higher with increasing education level of participants—primary (aPR = 1.27 [95% CI: 1.00–1.60]), secondary (ordinary level) (aPR = 1.47 [95% CI: 1.16–1.85]) and advanced level / tertiary (aPR = 1.55 [95% CI: 1.19–2.01]), and higher household income (aPR = 1.09 [95% CI: 1.00–1.20]). Additionally, participants who were not employed were less likely to have mosquito nets used in their households (aPR = 0.83 [95% CI: 0.70–0.98]). Houses that had undergone IRS in the previous 12 months were 42 (5.8%), while 220 (43.2%) households closed their windows before 6.00 pm. Environmental risk factors found at households included presence of vessels that could potentially hold water for mosquito breeding 414 (56.9%), and stagnant water in compounds 144 (19.8%). Several structural deficiencies on houses that could promote entry of mosquitoes were found such as lack of screening in ventilators 645 (94.7%), and external doors not fitting perfectly into walls hence potential for mosquito entry 305 (42.0%).

**Conclusion:**

There is need to increase coverage and utilisation of ITNs and IRS for malaria prevention in Wakiso district, Uganda. In addition, other malaria prevention strategies such as environmental management, and improving structural condition of houses are required to strengthen existing malaria prevention approaches.

## Introduction

Global malaria prevention efforts have focused on reducing the malaria burden mainly using insecticide-treated mosquito nets (ITNs) particularly long-lasting insecticidal nets (LLINs) and indoor residual spraying (IRS). These methods have been shown to reduce the occurrence of malaria in several studies [1,2]. The coverage of ITNs has increased markedly in recent years with several countries, mostly in Africa, distributing them free of charge [3]. Use of IRS remains low with only 2.9% of the global population at risk of malaria using it in 2016 [4], despite evidence on its efficacy in reducing malaria incidence. Furthermore, despite these proven preventive measures taken nationally and globally, the disease continues to cause severe morbidity and mortality particularly in sub-Saharan Africa. In 2016, there were 216 million cases of malaria and 445,000 deaths globally mainly occurring in Africa, and affecting children under 5 years of age [4].

Environmental risk factors associated with increased breeding of mosquitoes such as the presence of stagnant water and overgrown vegetation near homes are well known [5]. However, these factors have received little attention in the prevention of malaria despite recommended use of non-chemical and chemical methods of control in the context of integrated vector management [6]. The relatively inexpensive measures of removing pools of water, and clearing overgrown vegetation have been shown to significantly reduce mosquito abundance [7–9] and malaria incidence [10]. Such interventions can be used with core malaria prevention methods such as LLINs as a strategy to reduce occurrence of the disease. Indeed, to improve malaria prevention outcomes, it is important that interventions are implemented in a holistic manner. This calls for more research on integrated malaria prevention including environmental management [11].

Human exposure to malaria vectors in Africa mainly occurs indoors [12]. The structural design of houses is therefore critical in preventing entry of mosquitoes to reduce transmission of malaria [13]. Mosquitoes have for several years been known to normally enter houses through ventilators and open eaves [14], but also doors and windows [15]. Other openings on houses such as cracks in walls and broken window panes can also facilitate mosquito entry. Despite being known to protect against mosquitoes, the practice of screening houses has been largely ignored [16,17]. While assessing malaria prevention in communities, it is necessary to not only focus on core interventions such as ITNs and IRS, but also on conditions that contribute to presence of mosquitoes and their entry into houses. This study therefore assessed malaria prevention practices including not only ITNs and IRS, but also environmental risk factors around homes, and housing structure related to mosquito entry in a rural community in Wakiso district, Uganda.

## Methods

### Study area, context and sampling

The study was carried out in Ssisa sub-county, Wakiso district, in central Uganda; which is predominantly rural. All the 11 parishes in the sub-county were included in the study. Malaria is endemic in most parts of the country including Wakiso district. In Uganda, *Anopheles gambiae* is the predominant vector species responsible for transmitting malaria, whereas *Plasmodium falciparum* parasites are the leading cause of cases. The 2014–2015 malaria indicator survey established that nationally, malaria prevalence among children under 5 years was 18.9%, and 10.5% in the central region where Wakiso district is located [18]. The study clusters were the villages within each parish, with each village providing a minimum of 23 households. A total of 29 villages were included in the study, and 727 households were selected after taking into consideration clustering as described in our earlier paper [19]. Sampling proportionate to size was used to determine the number of villages to be selected from each parish, using current numbers of parishes and villages in the sub-county obtained from the Uganda Bureau of Statistics. The home of each village chairperson was used as the starting point during systematic sampling employed to select households per village involved in the study. The number of households in each village was used to determine the respective sampling interval.

### Study design and data collection

The study was a clustered cross-sectional survey that used quantitative data collection methods. Data were collected between 2014 and 2015 by trained research assistants using a questionnaire and observational checklist. The questionnaire collected data on participant demographics, malaria prevention practices used by households, number of mosquito nets owned by households including type, source and use the previous night. Data were collected about each net owned to a maximum of 3 nets per household. The observational checklist was used to assess environmental risk factors associated with occurrence of malaria present at households such as presence of stagnant water in compounds, and structural condition of houses related to mosquito entry such as lack of screening in ventilators. The questionnaire and checklist, which were pretested prior to data collected, were administered once for each household involved in the study. The study participants were household heads, and in their absence, another responsible adult found at home during data collection such as the spouse was involved.

### Data management and analysis

Data were entered in SPSS 10 (Chicago, Illinios, USA) and analysed in STATA 10 (College Station, Texas, USA). Univariate, bivariate and multivariate statistical data analysis procedures were followed. Practices on malaria prevention were assessed based on the methods that were being used by households. These methods were: sleeping under mosquito net; sleeping under ITN; taking preventive medicine; using body mosquito repellent; spraying house with insecticide; using mosquito coil; and removing mosquito breeding sites as used in the Uganda Malaria Indicator Survey 2009 [20]. Other methods assessed were IRS, closing of windows before 6.00pm, and removal of overgrown vegetation within 5 metres of houses. Assessment of structural condition of houses was done for the following parameters: windows having complete shutters or mosquito screening hence no space for possible mosquito entry; ventilators and open eaves having mosquito screening; houses with open eaves having ceilings; external doors having complete shutters and no space for possible mosquito entry; and presence of any other opening on house. The chi square test was used to assess association between households having pregnant women and their sleeping under mosquito nets. To ascertain the factors associated with use of mosquito nets, a generalized linear model with Poisson family, and a log link with robust standard errors was run where socio-demographic characteristics were analysed against use of mosquito nets (treated or untreated) that was coded as a binary outcome. Since the outcome variable was not rare, prevalence ratios (PRs) were preferred as the measure of association because odds ratios tend to overestimate the risk ratios in such instances [21]. Simple models were run to obtain the unadjusted PRs, then all variables were included in the multivariable model and a backward elimination method applied. A p-value of less than 0.05 was considered statistically significant.

### Ethical considerations

The study received ethical approval from Makerere University School of Public Health Higher Degrees, Research and Ethics Committee (123), and from the Uganda National Council for Science and Technology (SS 3294). Participants provided written informed consent after the purpose of the research had been clearly explained to them.

## Results

### Characteristics of participants and households

Of the 727 participants, 493 (67.8%) were female, 438 (60.3%) were aged 30 years or above, 329 (45.3%) had attained up to primary school education, and 78 (10.7%) had not attended any school. Further, 390 (53.7%) reported earning an equivalent of less than 40 US dollars per household per month; 347 (47.7%) households comprised of 3 to 5 members, and 238 (32.7%) of households had 2 or more children under 5 years of age. The majority of participants 416 (57.2%) were household heads (Table 1).

**Table 1:** Characteristics of participants and households in a rural community in Wakiso district, Uganda.

Variable	Frequency (N = 727)	Percentage (%)
**Gender**		
Female	493	67.8
Male	234	32.2
**Age (years)**		
18–29	289	39.8
≥ 30	438	60.3
**Highest level of education**		
None	78	10.7
Primary	329	45.3
Secondary (ordinary) level	259	35.6
Secondary (advanced) level / tertiary	61	8.4
**Occupation**		
Agriculture	235	32.3
Business	249	34.2
Housewife	82	11.3
Unemployed	108	14.9
Others (students, commercial motorcycle riders, fisher folk, stone miners)	53	7.3
**Average household monthly income (US dollars)**		
< 40	390	53.7
≥ 40	337	46.4
**Children under 5 years in household**		
None	254	34.9
1	235	32.3
≥ 2	238	32.7
**Position of participant in household in relation to household head**		
Household head	416	57.2
Spouse	227	31.2
Parent	35	4.8
Sibling	20	2.8
Other relative / not related	29	4.0
**Household size**		
1–2	112	15.4
3–5	347	47.7
≥ 6	268	36.9

### Practices on use of mosquito nets

Households that owned at least one mosquito net (ITN or untreated) were 471 (64.8%) with a mean number of nets owned of 2.6 (SD ± 1.9) compared to a mean household size of 5.0 (SD± 3.0). Most of the mosquito nets owned by households were LLINs particularly of *Permanet* brand 426 (44.5%), and provided by the Uganda Government 487 (50.8%). There was high use of the nets the night prior to collecting data 834 (87.1%), with the main reason for non-use being nets being too old / had many holes 54.0 (67%) (Table 2). There was a statistically significant association between households having pregnant women and use of mosquito nets by the same the night prior to the study (net 1 **χ**^**2**^ = 220.465, p < 0.001; net 2 **χ**^**2**^ = 57.415, p < 0.001; net 3 **χ**^**2**^ = 15.096; p = 0.001).

**Table 2:** Details of mosquito nets owned by households in a rural community in Wakiso district, Uganda.

Variable	Net 1 N = 471 (%)	Net 2 N = 311 (%)	Net 3 N = 176 (%)
**Source of net**			
Government	249 (52.9)	155 (49.8)	83 (47.2)
Shop / market / hawker	181 (38.4)	136 (43.7)	81 (46.0)
Other	29 (6.2)	10 (3.2)	6 (3.4)
Did not know	12 (2.5)	10 (3.2)	6 (3.4)
**Type of net**			
*Permanet*	217 (46.1)	132 (42.4)	77 (43.8)
*Duranet*	18 (3.8)	13 (4.2)	6 (3.4)
Other long lasting nets*	41 (8.7)	26 (8.4)	12 (6.8)
Non-long lasting nets**	9 (1.9)	5 (1.6)	3 (1.7)
Did not know	186 (39.5)	135 (43.4)	78 (44.3)
**Net was used the previous night**			
Yes	418 (88.7)	265 (85.2)	151 (85.8)
No	51 (10.8)	42 (13.5)	23 (13.1)
Not sure	2 (0.4)	4 (1.3)	2 (1.1)
**Reason for non-use of net**	**N = 53 (%)**	**N = 46 (%)**	**N = 25 (%)**
Net too old / had holes	31 (58.5)	23 (50.0)	13 (52.0)
Net not hang	17 (32.1)	19 (41.3)	9 (36.0)
Caused discomfort due to increased heat	4 (7.6)	1 (2.2)	1 (4.0)
Other reason	-	2 (4.3)	2 (8.0)
Did not know	1 (1.9)	1 (2.2)	-
**Number of people who used net the previous night**	**N = 471 (%)**	**N = 311 (%)**	**N = 176 (%)**
None	53 (11.3)	46 (14.8)	25 (14.2)
1	131 (27.8)	147 (47.3)	90 (51.1)
2	204 (43.3)	103 (33.1)	55 (31.3)
≥ 3	83 (17.6)	15 (4.8)	6 (3.4)
**Category of people who used net the previous night**	**N = 786 (%)**	**N = 407 (%)**	**N = 221 (%)**
< 5 years	248 (31.6)	135 (33.2)	56 (25.3)
Pregnant women	49 (6.2)	10 (2.5)	3 (1.4)
Others	489 (62.2)	262 (64.4)	162 (73.3)

* Other long lasting net brands were *Interceptor*, *Netprotect*, *Dawanet* and *Iconlife*.

** Non-long lasting net brands were *KO net*, *Kooper net*, *Iconet*, *Safi net*, *Century* and *Victoria*.

### Factors associated with use of mosquito nets by households

By bivariate analysis, use of mosquito nets was statistically associated with lower age of participants (unadjusted PR = 1.06 [95% CI: 0.96–1.67],) and education level–primary (unadjusted PR = 1.34 [95% CI: 1.07–1.69]), secondary (ordinary level) (unadjusted PR = 1.59 [95% CI: 1.27–1.99), and secondary (advanced) level / tertiary (unadjusted PR = 1.63 [95% CI: 1.27–2.01]). In addition, housewives (unadjusted PR = 1.19 [95% CI: 1.06–1.36]) and participants whose households had higher household income (unadjusted PR = 1.18 [95% CI: 1.08–1.29] had a higher prevalence of use of mosquito nets. In the multi variable model, participants who had received primary, secondary (ordinary level) and secondary (advanced level) / tertiary education were 27% (adjusted PR = 1.27 [95% CI: 1.00–1.60]), 47% (adjusted PR = 1.47 [95% CI: 1.16–1.85]) and 55% (adjusted PR = 1.55 [95% CI: 1.19–2.01]) more likely to use mosquito nets respectively compared to those who had no education. Furthermore, use of mosquito nets was lower among participants who were unemployed (adjusted PR = 0.83 [95% CI: 0.70–0.98]) while households that earned more than $40 monthly (adjusted PR = 1.09 [95% CI: 1.00–1.20]) were more likely to use mosquito nets compared to their counterparts (Table 3).

**Table 3:** Factors associated with use of mosquito nets by households in a rural community in Wakiso district, Uganda.

Variables	Use of mosquito nets	Unadjusted PR (95% CI)	p-value	Adjusted PR (95% CI)	p-value
**Overall**	530 (72.9)				
**Gender**					
Male	164 (70.1)	1		1	
Female	366 (74.2)	1.06 (0.96–1.67)	0.252	1.07 (0.96–1.18)	0.192
**Age (years)**					
18–29	224 (77.5)	1		1	
≥ 30	306 (69.7)	0.90 (0.82–0.98)	0.020*	0.96 (0.88–1.05)	0.356
**Highest level of education**					
None	40 (51.3)	1		1	
Primary	227 (69.0)	1.34 (1.07–1.69)	0.011*	1.27 (1.00–1.60)	0.048*
Secondary (ordinary level)	212 (81.8)	1.59 (1.27–1.99)	<0.001*	1.47 (1.16–1.85)	0.001*
Secondary (advanced level) / tertiary	51 (83.6)	1.63 (1.27–2.01)	<0.001*	1.55 (1.19–2.01)	0.001*
**Occupation**					
Agriculture	165 (70.2)	1		1	
Business	191 (76.7)	1.09 (0.98–1.22)	0.108	0.98 (0.88–1.09)	0.776
Housewife	69 (84.1)	1.19 (1.06–1.36)	0.005*	1.07 (0.94–1.22)	0.279
Unemployed	67 (62.0)	0.88 (0.74–1.05)	0.152	0.83 (0.70–0.98)	0.033*
Others	38 (71.7)	1.02 (0.84–1.23)	0.828	0.91 (0.75–1.10)	0.327
**Average household monthly income (US dollars)**					
≤ 40	262 (67.2)	1		1	
> 40	268 (79.5)	1.18 (1.08–1.29)	<0.001*	1.09 (1.00–1.20)	0.046*
**Religion**					
Christian	463 (73.1)	1			
Muslim	67 (71.3)	0.97 (0.85–1.12)	0.711	-	-
**Had children under 5 years in household**					
Yes	348 (73.6)	1			
No	182 (71.6)	1.02 (0.97–1.08)	0.435	-	-
**Household size**					
1–2	77 (68.7)	1			
3–5	257 (74.1)	1.08 (0.94–1.24)	0.296	-	-
≥ 6	196 (73.1)	1.06 (0.92–1.23)	0.402	-	-

* Statistically significant at p < 0.05

### Practices on other malaria prevention methods

Only 42 (5.8%) of the households had undergone IRS in the previous 12 months, among which 28 (66.7%) had been sprayed by household members. Other households were sprayed through government programmes 3 (7.1%), or private companies 7 (16.7%). Over half of the households that had been sprayed 22 (52.4%) paid for IRS. Among households that normally opened windows of their houses 509 (70%), 220 (43.2%) closed them before 6.00 pm. Only 129 (17.7%) of houses had ever been space sprayed with insecticides to kill mosquitoes. Among these houses, the frequency of space spraying was mainly whenever there was need 77 (59.7%). Other houses were space sprayed weekly 19 (14.7%), monthly 16 (12.4%) or fortnightly 8 (6.2%). Other malaria prevention methods used by households were removing mosquito breeding sites 181 (24.9%), using mosquito coils 70 (9.6%), and taking preventive medicine 20 (2.8%). A total of 105 households (14.4%) were doing nothing to prevent malaria.

### Environmental risk factors related to malaria, and structural condition of houses

Environmental factors that favour mosquito breeding found at households included presence of vessels in the compound that could potentially hold water 414 (56.9%) and stagnant water in compounds 144 (19.8%). Several structural deficiencies on houses that could promote entry of mosquitoes were found such as lack of screening in ventilators 645 (94.7%), external doors not fitting perfectly into the walls hence potential for mosquito entry 305 (42.0%), and presence of other openings on houses where mosquitoes could pass 265 (36.5%) (Table 4).

**Table 4:** Environmental risk factors, and structural condition of houses in a rural community in Wakiso district, Uganda.

Risk factor / structural condition	Frequencies	Percentage (%)
**Environmental risk factors**		
Stagnant water present in compound (N = 727)	144	19.8
Presence of vessels that could potentially hold water for mosquito breeding (N = 727)	414	56.9
Presence of overgrown vegetation within 5 metres of houses (N = 727)	555	76.3
**Structural condition of houses**		
Houses with windows lacking complete shutters (N = 727)	192	26.4
Houses with windows not fitting perfectly into wall (with space for possible mosquito entry) (N = 727)	244	33.6
Houses lacking screening in windows to prevent mosquito entry (N = 727)	698	96.0
Windows found open lacking screening to prevent mosquito entry (N = 371)	345	93.0
Houses with ventilators lacking screening to prevent mosquito entry (N = 681)	645	94.7
Houses with open eaves lacking complete screening to prevent mosquito entry (N = 194)	190	97.9
Houses with open eaves lacking ceilings (N = 194)	179	92.3
External doors lacking complete shutters (N = 727)	160	22.0
External doors not fitting perfectly into walls (with space for possible mosquito entry) (N = 727)	305	42.0
Other opening on house (such as hole in wall) where mosquitoes could pass to enter house (N = 727)	265	36.5

## Discussion

Our study established that generally, there was low ownership and use of various malaria prevention methods including LLINs and IRS. Use of mosquito nets by households increased with education of participants (who were mainly household heads), and household income. However, participants who were not employed were less likely to have mosquito nets used in their households to prevent malaria. In addition, several risk factors that promote presence of mosquitoes at households existed in the community such as potential breeding sites. It was also established that the structural condition of houses in the community enhanced mosquito entry including lack of screening in ventilators. The findings of the study therefore emphasize the fact that vast community practices (or lack of them) still predispose the population to the disease that causes severe morbidity and mortality in the country [18].

Ownership of at least one mosquito net by households was relatively high (64.8%) despite being much lower than the current national figure of 90% who owned an ITN [18]. However, it is evident that the number of mosquito nets owned was not sufficient for members of the households with a mean number of nets of 2.6 compared to a mean household size of 5.0. Although use of available nets the night prior to the study was high (86.6%), many individuals are likely to be exposed to mosquito bites due to the insufficient number of nets in households. Since use of ITNs is the most advocated method for malaria prevention globally [4], there is need for its increased coverage and utilisation. One of the barriers identified for the low ownership of mosquito nets particularly in low income countries is the high cost of buying them [22]. Indeed, most of the nets owned by households in this study were provided by the government free of charge. These nets have previously been distributed by Ministry of Health mainly targeting pregnant women and children under 5 years of age through mass campaigns and antenatal visits [23]. Such campaigns are likely to be responsible for the association between households having pregnant women and their sleeping under mosquito nets found in this study. More recently, other household members beyond children and pregnant women have benefitted from government campaigns of receiving free nets. Indeed, the national increase in coverage of LLINs can be partly attributed to the government efforts of availing them to the population in recent years [18].

Our study established that use of mosquito nets by households increased with increasing education level of household heads. In addition, participants who were not employed were less likely to have mosquito nets used in their households. Other studies carried out in sub-Saharan Africa have also established use of mosquito nets to be associated with education, income and employment [24,25,26]. Highly educated individuals would normally be aware of core malaria prevention practices including use of mosquito nets hence increased use in their households. In addition, households with higher income are more likely to spend on malaria prevention including purchase of mosquito nets in comparison with their counterparts. Given that employment is directly related to income, it was logical to establish in our study that unemployment had a negative effect on use of mosquito nets in households. Practices on malaria prevention particularly use of mosquito nets are therefore likely to improve with increased education, employment and income among the population as established in our study.

Although IRS is a key global method for malaria prevention, its use in the study was low (5.8%). Studies done in other malaria endemic countries have also found low coverage of IRS [27,28,29]. Community acceptance of IRS has been impeded by insecticide smell, mess left by the sprayers, inconvenience of removing household items from houses before spraying, increased prevalence of other insects, perceived ineffectiveness, and side effects [30,31,32]. These barriers need to be addressed so as to increase utilisation of IRS for malaria prevention. Use of insecticide space sprays was also found to be low in this study (17.7%) as was the case in other studies in sub-Saharan Africa [16,33,34]. Such sprays are known to be costly, hence may not be used by poor populations including in Uganda. In addition, there are concerns about the potential impact on health and the environment that they might cause [35]. In this study, less than half of households (43.2%) that normally opened windows on their houses closed them before 6.00 pm. As endophagic malaria transmitting mosquitoes have for long been known to enter houses in the early hours of the evening [36, 37], it is ideal that windows should be closed before that time [38]. However, this simple practice of closing windows at an appropriate time to limit mosquito entry into houses is apparently ignored in many communities. Therefore, there is need by various stakeholders such as health practitioners and community health workers to promote early closing of windows (and doors) among communities to reduce mosquito entry into houses.

Malaria transmitting mosquitoes are known to breed in pools of water [39] and habour in vegetation [5], both often found near houses especially in rural communities in endemic countries. This study found that 56.9% of houses had vessels in their compounds that could potentially hold water for mosquito breeding, and 76.3% had overgrown vegetation within 5 metres. This is an indication that conditions that support mosquito breeding are allowed to exist in communities. Although environmental management practices such as removing mosquito breeding sites have shown promise in vector control, they have often received little attention by malaria control stakeholders [40]. Given that the burden of malaria remains high particularly in sub-Saharan Africa even after extensively promoting use of LLINs and IRS for many years, it is prudent that other malaria prevention strategies such as removal of potential mosquito breeding sites notably stagnant water are widely promoted to complement existing ones. Indeed, a holistic approach to malaria prevention encompassing several strategies and interventions is likely to have greater public health benefits.

Malaria transmitting mosquitoes particularly in sub-Saharan African mainly bite humans indoors at night [12] hence enter houses through openings such as ventilators and open eaves [41]. In many rural communities in malaria endemic countries, houses have poor structure that promote entry of mosquitoes hence malaria transmission [13,42]. Indeed, this study found several structural defects on houses such as lack of screening in ventilators (94.7%) and open eaves (97.9%), and external doors not fitting perfectly into the wall hence having space for possible mosquito entry (42.0%). Although houses with open eaves but have ceilings would observe reduced mosquito entry [43], 92.3% of such houses in this study lacked them. Screened houses and those with closed eaves have been found with reduced incidence of malaria among occupants [44]. In addition, screening of houses not only protects all household members from malaria but also other mosquito related and vector borne diseases transmitted indoors such as yellow fever and dengue. It is therefore important that the structural condition of houses is appropriate to reduce mosquito entry. This is particularly important in communities with low ownership and use of ITNs and other core malaria prevention methods.

A limitation of this study is that although both treated and untreated mosquito nets were assessed, ITNs are recommended by the World Health Organization [45] because of their ability to not only prevent mosquito bites but also kill mosquitoes. In addition, LLINs retain their biological activity for a long period hence can be used for up to three years without retreatment [46]. Nevertheless, even untreated mosquito nets provide a protective barrier against mosquitoes which alone is a prevention method. In addition, as most of the nets were received from the government which provided ITNs, the non-treated nets were likely to be minimal. Challenges have also been realised in distinguishing untreated nets from ITNs [47] and observing those already hang in houses is often impractical. One strength of the study is that the environmental conditions at households as well as the structural conditions of houses were observed with help of a checklist hence eliminating social-desirability bias usually associated with questionnaire surveys.

## Conclusions

Although mosquito nets were predominantly used, there is need to increase coverage and utilisation of ITNs and IRS for malaria prevention with attention given to barriers such as education and income. In addition, other malaria prevention strategies such as environmental management and early closing of windows, as well as improving structural condition of houses need to be strengthened to complement existing malaria prevention approaches in endemic countries including Uganda.

## Supporting information

S1 Dataset This is the study dataset. (DTA)
